# Survival outcomes in HER2-low versus HER2-zero breast cancer after neoadjuvant chemotherapy: a meta-analysis

**DOI:** 10.1186/s12957-024-03382-w

**Published:** 2024-04-20

**Authors:** Lin-Yu Xia, Xu-Chen Cao, Yue Yu

**Affiliations:** 1 The First Department of Breast Cancer, Tianjin Medical University Cancer Institute and Hospital, National Clinical Research Center for Cancer, 150 meters north of the intersection of Xinjiayuan North Road and Xinjin Road Xinjin Road, Binhai New District, Tianjin, 300060 China; 2 Key Laboratory of Breast Cancer Prevention and Therapy, Tianjin Medical University, Ministry of Education, Tianjin, 300060 China; 3 Key Laboratory of Cancer Prevention and Therapy, Tianjin, 300060 China; 4 Tianjin’s Clinical Research Center for Cancer, Tianjin, 300060 China

**Keywords:** Breast cancer, HER2-low, HER2-zero, Neoadjuvant chemotherapy

## Abstract

**Background:**

The survival outcomes in HER2-low versus HER2-zero breast cancer (BC) after neoadjuvant chemotherapy (NACT) remain unclear. The meta-analysis was conducted to summarize current evidence about the survival outcomes in HER2-low versus HER2-zero BC.

**Methods:**

We conducted a systematic search in PubMed and EMBASE databases to identify relevant studies.

**Results:**

A total of 14 studies with 53,714 patients were included. Overall, 34,037 patients (63.37%) were HER2-low, and 19,677 patients (36.63%) were HER2-zero. Patients with HER2-low tumors had a significantly lower pathological complete response (pCR) rate than patients with HER2-zero tumors, regardless of the hormone receptor status. Compared with HER2-zero breast cancer, the overall survival (OS) and disease-free survival (DFS) of HER2-low BC were longer in the overall cohort (HR = 0.72; 95% CI = 0.61–0.85; *P* < 0.0001; HR = 0.83; 95% CI = 0.75–0.92; *P* = 0.0002); however, no differences were observed in terms of OS and DFS between HER2-low and HER2-zero BC in the HR-negative group. In the HR-positive group, HER2-low status had no significant impact on OS, while significantly associated with increased DFS (HR = 0.85; 95% CI = 0.76–0.96; *P* = 0.007).

**Conclusion:**

These results suggest that although HER2-low BC has a poor response to NACT, it is correlated with favorable OS and DFS after NACT in the overall cohort as well as longer DFS in the HR-positive group.

**Supplementary Information:**

The online version contains supplementary material available at 10.1186/s12957-024-03382-w.

## Introduction

Human epidermal growth factor receptor 2 (HER2) is a receptor for transmembrane tyrosine kinases, which is directly related to the aggressive growth of BC and is an important target for BC treatment [1]. Trastuzumab is the first approved monoclonal antibody against HER2, which can target the extracellular domain of HER2 protein. H0648g, BCIRG 007, and other studies showed that trastuzumab combined with chemotherapy significantly prolonged the survival time of BC patients with HER2 overexpression (IHC 3 + or IHC 2 + with ISH positivity) [2, 3]. Studies such as NSABP B-47/NRG confirmed that patients with low or moderate HER2 expression (IHC 1+, or 2+/ISH negative) cannot benefit from traditional targeted drugs [4, 5]. Therefore, the HER2 status has always been divided into two categories: HER2 low or moderate expression and HER2 zero expression are classified as HER2 negative (IHC 0, 1+, or 2+/ISH negative), while HER2 overexpression (IHC 3 + or IHC 2 + with ISH positive) is classified as HER2 positive [6]. However, the recent development of novel antibody-drug conjugates (ADCs) has significantly improved the prognosis of BC patients with low or moderate HER2 expression (1+, or 2+/ISH negative), thus leading to the concept of “HER2-low breast cancer” [7, 8].

Currently, most studies define BC with low or moderate HER2 expression (IHC 1+, or 2+/ISH negative) as HER2-low BC [9–12]. Based on the fact that HER2-low BC has low or moderate HER2 expression and can benefit from new targeted drugs, some scholars proposed that HER2-low BC is different from HER2-zero, that is, different from luminal BC or triple-negative BC, and may be an independent subtype. This has aroused strong interest among researchers. It is currently known that HER2-low BC has a large population, accounting for approximately 40–50% of breast cancers [13]. The biological, clinicopathological, and prognostic differences between HER2-low and HER2-zero breast cancers have been reported [9, 10, 14, 15]. However, HER2-low BC has not yet been established as an independent subtype. Although novel targeted drugs have brought benefits to HER2-low BC, they have not yet been approved for front-line treatment of non-metastatic HER2-low BC. Chemotherapy remains one of the most important treatments for non-metastatic HER2-low BC, especially for HER2-low BC patients with HR-negative or HR-positive who are resistant to endocrine therapy. Exploring the differences in chemotherapy sensitivity and prognosis between HER2-low BC and HER2-zero BC can help us further discover the differences between HER2-low BC and HER2-zero BC, and also help us better understand the clinicopathological characteristics of HER2-low BC and its sensitivity to chemotherapy, to provide a basis for the later formulation of HER2-low BC treatment plan. However, multiple studies have reached different conclusions about the effects of HER2-low and HER2-zero on the response and prognosis of neoadjuvant chemotherapy [11, 16–20]. Given the conflicting conclusions, we conducted this meta-analysis to compare the survival outcomes in HER2-low versus HER2-zero BC after neoadjuvant chemotherapy.

## Materials and methods

### Search strategy

This meta-analysis was conducted strictly following the PRISMA 2020 statement [21]. The PRISMA checklist is shown in Additional File 1. We performed a systematic literature search in the PubMed and Embase databases for studies published by October, 2023. Keywords used were; (“breast cancer, OR breast neoplasm” AND “HER2-low”). Any geographical region or language was accepted. The detailed reproducible search strategy for each of the databases is shown in Additional File 2.

### Inclusion and exclusion criteria

The published studies were to meet the following inclusion criteria; (1) BC patients diagnosed with HER2-low or HER2-zero; (2) patients treated with NACT and surgery; (3) HER2-low was defined as HER2 IHC 1 + or 2+/ISH negative. (4) the study must have reported the pCR rate and the survival outcomes in terms of OS and/or DFS; (5) the study compared the survival outcomes between HER2-low and HER2-zero. Exclusion criteria were as follows; (1) review articles, letters to the editor, comments, editorials, and case reports; (2) patients with metastatic disease or other malignant tumors; (3) Lacking data on clinical outcome that could be used to calculate the HRs and 95% CIs.

### Study selection and data extraction

We selected the studies according to the search strategy and inclusion and exclusion criteria. The standardized data extraction form was used to extract the relevant information such as the first author’s name, year of publication, study design, nationality and the number of patients, the median age of participants, the tumor stage and histology, Nottingham grade, local treatment, median follow-up time, survival outcomes.

### Quality assessment

The study quality was assessed based on eight items from the non-randomized experimental research-MINORS scale [22], Each item was scored as 0 (not reported), 1 (inadequately reported), or 2 (adequately reported). we only retained studies with scores of 8 or more, which were rated as high-quality (See Supplementary Table 2, Additional File 3).

### Summary measures and statistical analysis

We used RevMan version 5.3 (RevMan, version 5.3 for Windows; Cochrane Collaboration, Oxford, UK) for Meta-analysis. The hazard ratios (HRs) were extracted from published.

data or Kaplan-Meier survival curves. Statistical heterogeneity was assessed by Chi-squared and I^2^. When I^2^ > 50%, the test of heterogeneity was significant, thus, the random-effects model was used; otherwise, the fixed-effects model was used [23]. Funnel plot and Begg’s test were used to assess the potential publication bias [24]. They were performed with the Stata Version 11.0 (Stata Corporation, College Station, TX, USA). All tests were two-sided. *P* < 0.05 was considered statistically significant.

## Results

### Study selection and characteristics

**Fig. 1:**
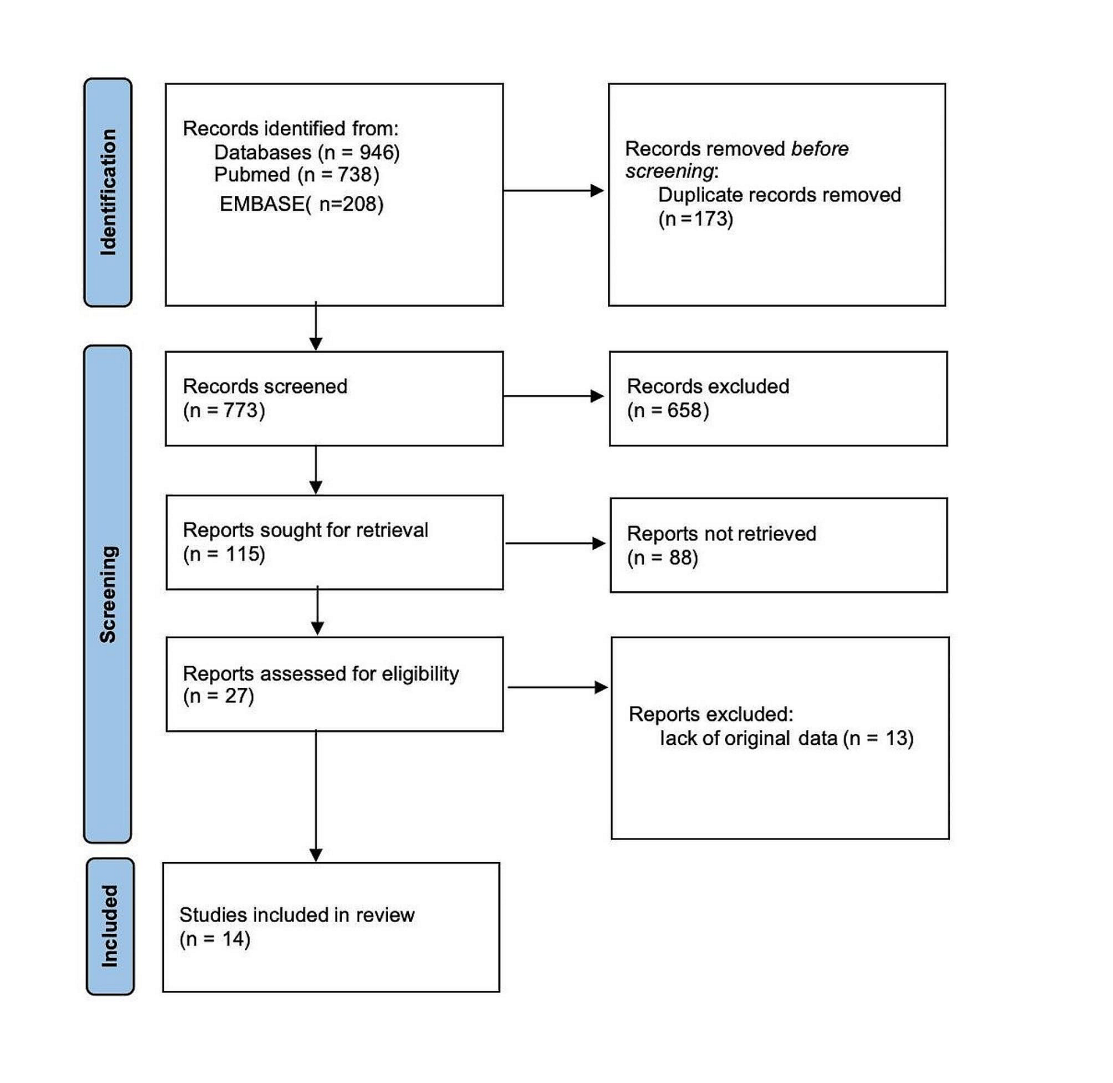
Flowchart explaining the article selection

A total of 946 articles were identified. 773 articles were retained after removing duplicates. After reviewing the title and abstract, 658 articles irrelevant to this study were also removed. 13 articles were excluded after the full text was reviewed according to the inclusion and exclusion criteria. In the end, 14 studies with 53,714 patients who meet the criteria were included [11, 13, 16–20, 25–31]. As shown in Fig. 1.

All the included studies were retrospective cohort studies published in 2021 to 2023. In the studies, 34,037 patients (63.37%) were HER2-low, and 19,677 patients (36.63%) were HER2-zero. 10 studies reported that 21,190 (67.11%) of 31,574 patients with HER2-low were HR-positive, compared with 8753 (46.98%) of 18,631 patients with HER2-zero. The information on characteristics is shown in Tables 1 and 2.

**Table 1:** Main characteristics of the eligible studies

First author	Year	country	Study type	Participations(n) (HER2-low/ HER2-zero)	Clinical stage	Follow-up (median) (month)	HR estimation	Outcomes (All)	Outcomes (HR+)	Outcomes(HR-)
Denkert	2021	Germany	retrospective	2310 (1098/1212)	I–III	46.6	Given by author	OS, DFS	OS, DFS	OS, DFS
de Moura	2021	South America	retrospective	855 (285/570)	I–III	59	Survival curve	OS	OS	OS
Domergue	2022	France	retrospective	437 (121/316)	I–III	72.9	Survival curve	NR	NR	OS, DFS
Alves	2022	Portugal	retrospective	72 (41/31)	II–III	35.5	Survival curve	OS, DFS	NR	NR
Cosimo	2022	Italy	retrospective	444 (335/109)	I–III	59.6	Survival curve	DFS	DFS	DFS
Shao	2022	China	retrospective	314 (226/88)	II–III	36	Survival curve	OS, DFS	OS, DFS	OS, DFS
Kang	2022	Korea	retrospective	1572 (754/818)	I–III	NR	Given by author	OS, DFS	OS, DFS	OS, DFS
Zhou	2023	China	retrospective	325 (234/91)	I–III	29.3	Given by author	OS, DFS	NR	NR
Qiao	2023	China	retrospective	132 (70/62)	II–III	20	Survival curve	OS, DFS	OS, DFS	OS, DFS
Li	2023	China	retrospective	1027 (678/349)	I–III	56	Survival curve	OS, DFS	OS, DFS	OS, DFS
Pöschke	2023	Germany	retrospective	1373 (930/443)	I–III	120	Survival curve	OS, DFS	OS, DFS	OS, DFS
Li JJ	2023	China	retrospective	283 (239/44)	II–III	59	Survival curve	OS	OS	OS
Zhong	2023	US	retrospective	41,500 (26,686/14,814)	I–III	NR	Given by author	OS	OS	OS
Zhang	2023	China	retrospective	3070 (2340/730)	I–III	40	Survival curve	NR	OS, DFS	NR

NR = not reported

**Table 2:** Patient and tumor characteristics of the the eligible studies

First author	Participations(n)		median age(Y)	Clinical stage		Histologic grade		Histology		Local treatment	
	HER2-low	HER2-zero	HER2-low/ HER2-zero	HER2-low	HER2-zero	HER2-low	HER2-zero	HER2-low	HER2-zero	HER2-low	HER2-zero
	HR+/HR-	HR+/HR-		I/II/III	I/II/III	1/2/3	1/2/3	IDC/ILC/Other	IDC/ILC/Other	M/B	M/B
Denkert	703/395	445/767	49/48	NR	NR	24/417/657	15/322/875	927/45/126	1032/27/153	NR	NR
de Moura	236/49	306/264	45.66/45.07	1/110/176	12/233/325	26/148/100	24/255/268	246/18/21	472/49/49	219/66	401/169
Domergue	0/285	0/570	52.0/51.0	NR	NR	NR	NR	112/3/6	304/4/8	50/76	107/216
Alves	29/12	11/20	53/50	0/14/27	0/19/12	9/19/13	1/17/13	31/6/4	26/3/2	32/9	20/11
Cosimo	272/63	47/62	49/51.2	NR	NR	14/145/140	1/28/61	NR	NR	229/106	60/49
Shao	171/55	56/32	47/47	0/108/118	0/38/50	NR	NR	NR	NR	182/32	74/10
Kang	611/143	458/360	46/46	NR	NR	9/586/155	11/553/251	707/23/24	757/25/36	NR	NR
Zhou	147/87	37/54	47/46	2/88/140	1/37/49	19/105/55	1/29/31	6/206/22	4/81/6	NR	NR
Qiao	53/17	23/39	NR	0/57/13	0/51/11	NR	NR	NR	NR	61/9	58/4
Li	426/252	138/211	NR	29/507/142	21/258/70	NR	NR	608/48/22	298/40/11	579/77	300/32
Pöschke	661/267	235/208	NR	NR	NR	32/351/544	20/156/260	666/78/185	302/46/92	331/599	161/282
Li JJ	188/51	19/25	NR	0/96/143	0/17/27	NR	NR	NR	NR	231/8	41/3
Zhong	17,693/8993	6978/7836	NR	NR	NR	NR	NR	NR	NR	NR	NR
Zhang	NR	NR	NR	NR	NR	NR	NR	NR	NR	NR	NR

NR = not reported

### Pathologic complete response

**Fig. 2:**
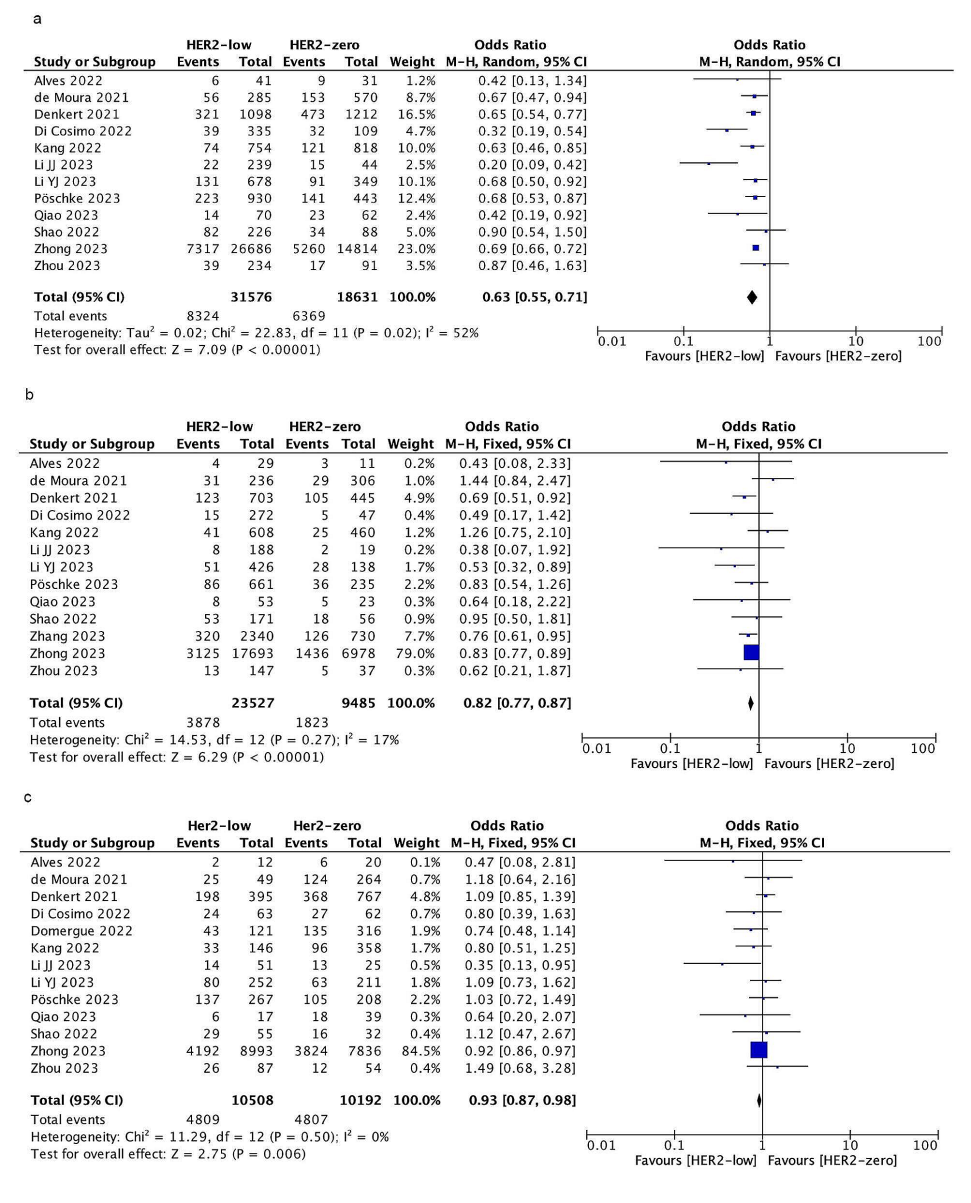
Forest plot of the OR for the pCR rate of HER2-low breast cancer vs. HER2-zero breast cancer in overall cohort (**a**), HR-positive group(**b**), HR-negative group(**c**)

8324 out of 31,576 patients with HER2-low achieved pCR, while 6369 out of 18,631 patients with HER2-zero achieved pCR. HER2-low patients had a significantly lower pCR rate than HER2-zero patients (26.36% VS. 34.18%, OR = 0.63; 95% CI = 0.55–0.71; *P* < 0.00001). Heterogeneity was detected among these data (I ^2^ = 52%, *P* = 0.02). According to hormone receptor status, the pCR rate of HER2-low patients was also lower than that of HER2-zero patients in HR-positive group (16.48% vs. 19.22.00%, OR = 0.82; 95% CI = 0.77–0.87; *P* < 0.00001) and HR-negative group(45.77% vs. 47.16%, OR = 0.93; 95% CI = 0.87–0.98; *P* = 0.006). There was no heterogeneity in the two subgroups. As shown in Fig. 2.

### Overall survival

**Fig. 3:**
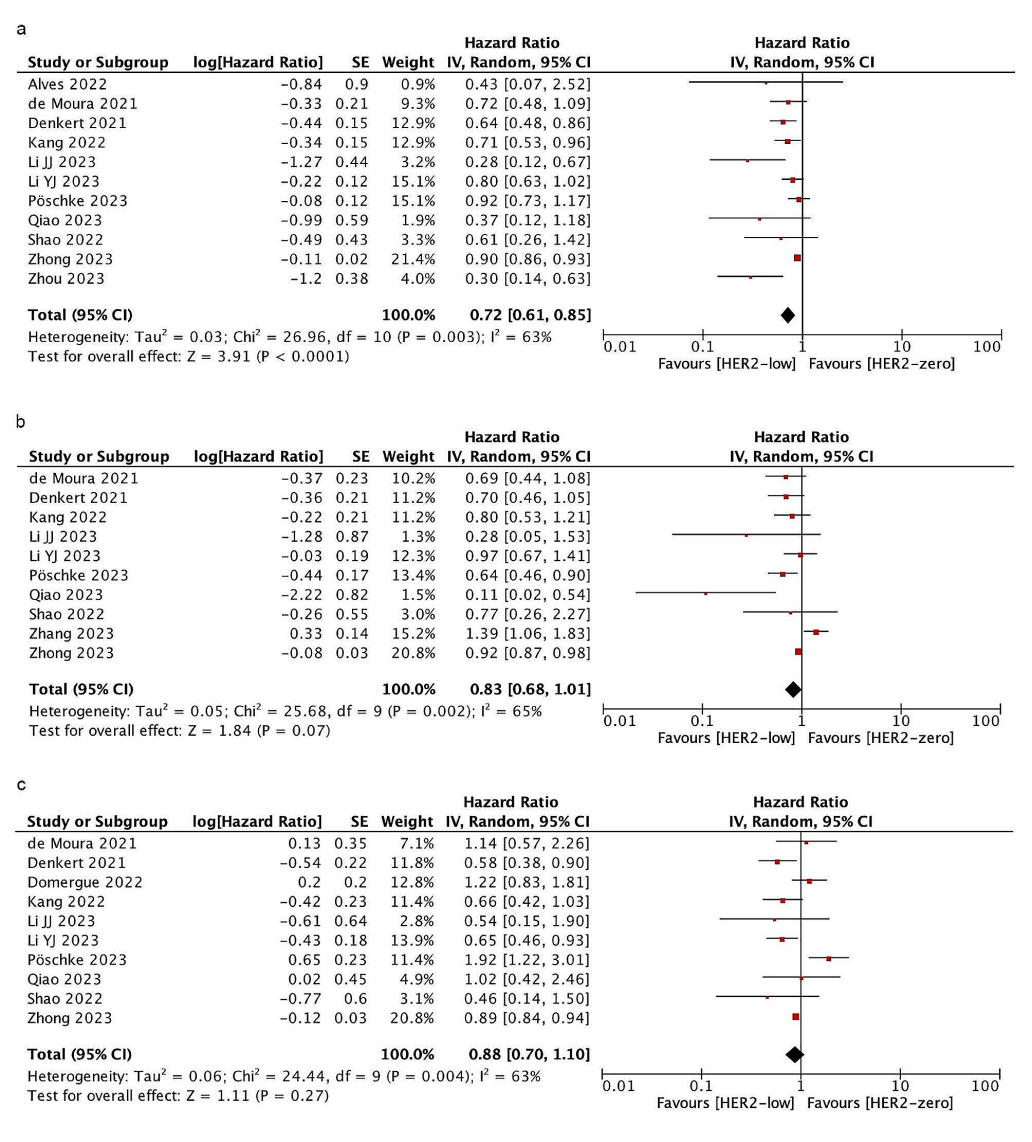
Forest plot of the HR for OS of HER2-low breast cancer vs. HER2-zero breast cancer in overall cohort (**a**), HR-positive group(**b**), HR-negative group(**c**)

Eleven studies involving 49,763 people reported OS. After a median follow-up of 46.6 months, HER2-low patients showed a longer OS than HER2-zero (HR = 0.72; 95% CI = 0.61–0.85; *P* < 0.0001). In the subgroup analysis, there was no significant difference in OS between HER2-low and HER2-zero patients in the HR-positive group (HR = 0.83; 95% CI = 0.68–1.01; *P* = 0.07) and HR-negative group (HR = 0.88; 95% CI = 0.70–1.10; *P* = 0.27). Significant heterogeneity existed among the studies. As shown in Fig. 3.

### Disease-free survival

**Fig. 4:**
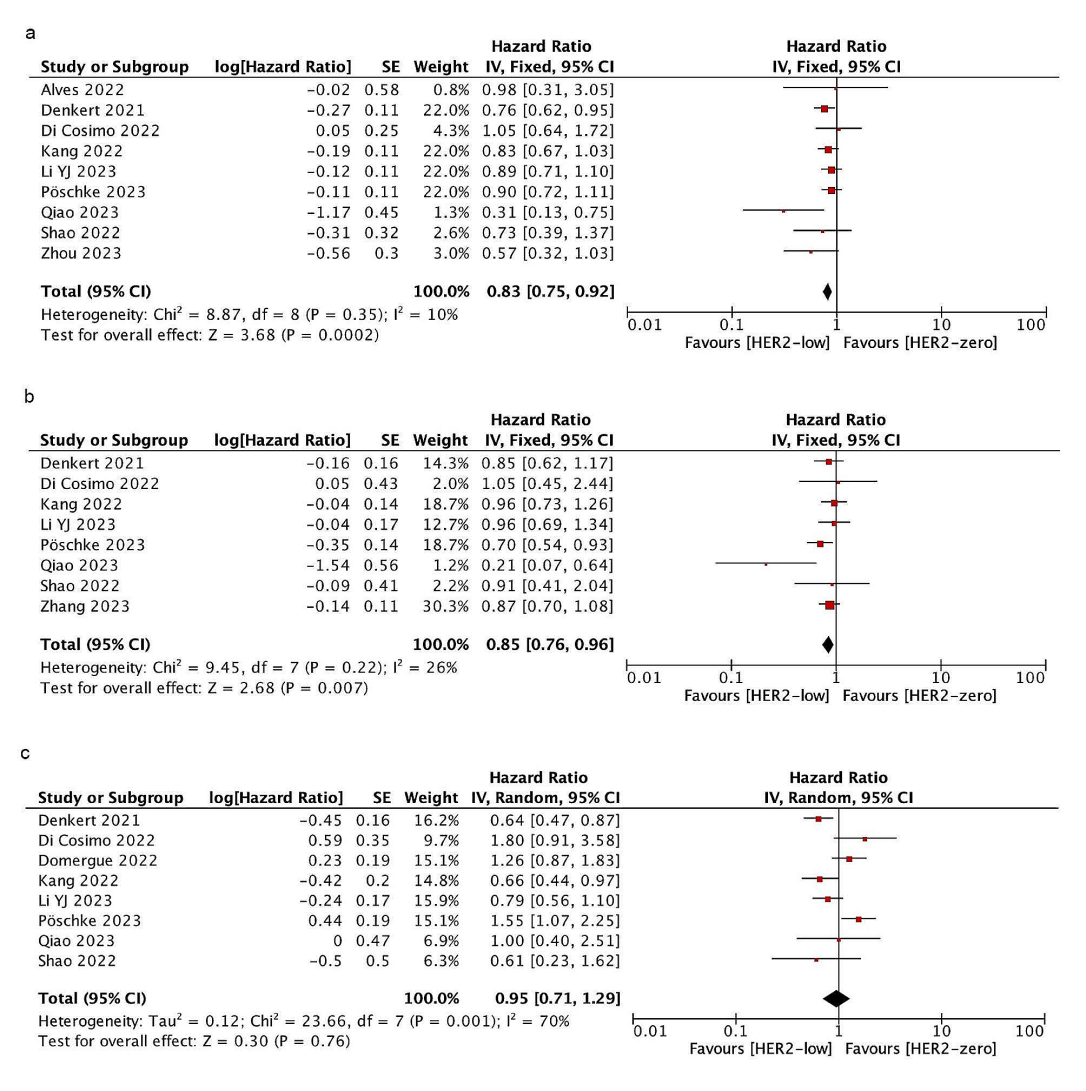
Forest plot of the HR for DFS of HER2-low breast cancer vs. HER2-zero breast cancer in overall cohort (**a**), HR-positive group(**b**), HR-negative group(**c**)

Nine studies involving 7569 people reported DFS. After a median follow-up of 35.75 months, the DFS of HER2-low patients is significantly better than that of HER2-zero patients (HR = 0.83; 95% CI = 0.75–0.92; *P* = 0.0002). According to hormone receptor status, this survival trend was also true in the subgroups of patients with HR-positive tumors (HR = 0.85; 95% CI = 0.76–0.96; *P* = 0.007). However, no survival difference was seen between them in HR-negative tumors (HR = 0.95; 95% CI = 0.71–1.29; *P* = 0.76). Heterogeneity only exists in the HR-negative group. As shown in Fig. 4.

**Fig. 5:**
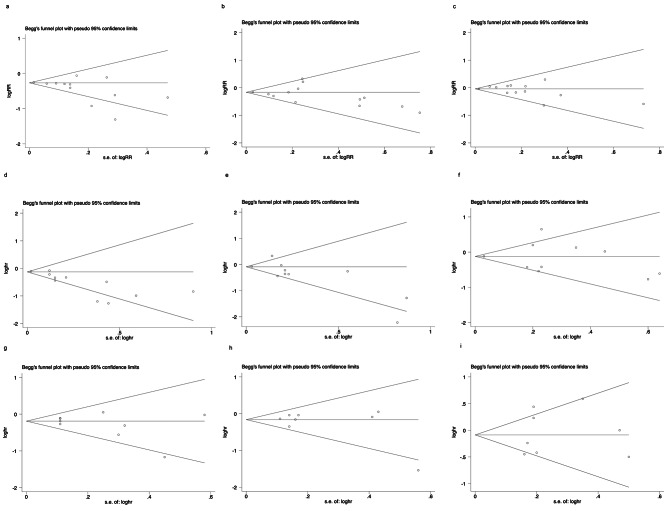
Funnel plot of HER2-low breast cancer vs. HER2-zero breast cancer. OR for the pCR rate: overall cohort (**a**), HR-positive group(**b**), HR-negative group(**c**); HR for OS: overall cohort (**d**), HR-positive group(**e**), HR-negative group(**f**); HR for DFS: overall cohort (**g**), HR-positive group(**h**), HR-negative group(**i**)

The funnel plots and Begg’s test were used to detect the publication bias ( Fig. 5). All *P* values were > 0.05 ( See Supplementary Table 3, Additional File 4), suggesting no potential publication bias was found in the pCR rate, OS and DFS.

## Discussion

HER2-low BC is now increasingly considered a distinct subtype. We analyzed the outcomes of HER2-low BC through 34,037 patients in 14 studies. A lower rate of pCR was observed in the patients with HER2-low versus those with HER2-zero, regardless of the hormone receptor status. HER2-low patients had superior OS and DFS compared to HER2-zero patients in the overall group. According to hormone receptor status, HER2-low patients led to a better DFS in the HR-positive group, while no significant survival difference was seen between them in other groups.

Previous studies reported that the proportion of HER2-low in HER2-negative ranged from 33.3 to 72.1% [13, 32–34]. Our study shows that the proportion is 63.37%. The difference in proportion between different studies may be due to the limitations of current detection and interpretation of HER2 expression. At present, the difficulties and inconsistencies mainly focus on the interpretation of 0 and 1+. According to the American Society of Clinical Oncology(ASCO) guideline: IHC 0: no staining or ≤ 10% of cells with incomplete membrane staining that is faint / barely perceived. IHC 1+: >10% of cells with incomplete membrane staining that is faint/barely perceptible [6]. Patients with 1 + have weak staining and need to be observed more closely. The interpretation of human eyes is subjective and inconsistent. Previously, the treatment of HER2 (1+) was the same as HER2-zero, so the pathologist himself may not pay much attention to the distinction between HER2 immunohistochemistry 0 and 1+. Secondly, in the detection process, the detection antibodies and platforms of each pathology department are different, which may also lead to differences in the determination. In 2023, ASCO guidelines specifically proposed 5 recommendations to distinguish IHC 1 + results from 0 in the update of HER2 testing guidelines. For example, examining HER2 IHC at high power (40×) when discriminating 0 from 1 + staining and considering a second pathologist review when results are close to the 0 versus 1 + interpretive threshold, and so on [35]. Various studies on how to achieve accurate detection of HER2 expression are also being tried. For example, droplet microfluidic technology [36], analysis of HER2 messenger RNA levels [37, 38], and digital pathology achieved through artificial intelligence [39, 40].

The results of this study showed that Luminal BC accounted for the majority of HER2-low patients, and the pCR rate of HER2-low patients was lower than that of HER2-zero. This is consistent with previous research. Schettini et al. studied the expression of PAM50 and individual gene expression in 1,320 patients (35.8%) and found that about 65% of HER2-low patients were HR-positive compared with the HER2-zero group. In the overall cohort, the expression of relative proliferation-related genes is significantly down-regulated and the expression of luminal-related genes is up-regulated in HER2-low patients, while most proliferation-related genes and tyrosine kinase receptor genes are more expressed in HER2-zero tumors. In the HR-positive subgroup, similar gene expression differences were observed. However, in the HR-negative subgroup, no differential gene expression was found between HER2-low and HER2-zero [9]. Zhang et al. also found that 87.5% of the HER2-low subgroup were luminal tumors [41]. The low sensitivity of luminal tumors to chemotherapy may explain the low pCR rate in the HER2-low subgroup. It is interesting that in our study, even in the HR-negative subgroup, the pCR rate of HER2-low patients was lower than that of HER2-zero. Previous studies have found that compared with HER2-zero tumors, HER2-low tumors tend to have lower ki-67 expression and lower histological grade [16, 27, 41]. Dehghani et al. analyzed HER2-low and HER2-zero patients in triple-negative BC and found that HER2-low tumors had lower lymph node involvement rates, less lymphatic invasion, and lower local recurrence rates [42]. So we speculate that even though there is no difference in gene expression between the two groups in the HR-negative subgroup, HER2-low tumors still have low invasiveness, reducing their sensitivity to chemotherapy.

Our analysis shows that HER2-low tumors are better than HER2-zero in both OS and DFS in the overall cohort. Given the role of HER2 in the pathogenesis of breast cancer, we believe that even its expression at low levels would be associated with more aggressive characteristics than its complete absence. However, as mentioned earlier, HER2-low tumors are less aggressive than HER2-zero tumors, both in terms of gene expression and pathological features. This may be related to the high expression of hormone receptors in HER2-low tumors. Most studies have proven that there is an interaction between the signaling pathways of HER2 and hormone receptors. ER signaling can down-regulate HER2 expression, which has a significant impact on HER2-low expression and related tumor biology [43, 44]. The low invasiveness of HER2-low tumors may be the reason for their better prognosis. However, in subgroup analysis, HER2-low had an advantage over HER2-zero in DFS only in the HR-positive group, and no significant differences were noted for OS or DFS in other groups. We speculate that the reason may be related to the low pCR of HER2-low breast cancer. It is possible that the negative impact of low pCR on survival outweighs the positive effect of low tumor aggressiveness on survival in these subgroups.

Traditional anti-HER2 therapies do not benefit HER2-low BC patients [5]. In recent years, HER2-targeted antibody-drug conjugates (ADCs): trastuzumab deruxtecan (T-DXd) and trastuzumab duocarmazine (SYD985) have shown promising anti-tumor activity in patients with HER2-low BC [45, 46]. The results of DESTINY Breast-04 showed that trastuzumab deruxtecan (T-Dxd) significantly improved the objective response rate (52.3% VS. 16.3%) in patients with previously extensively treated HER2-low advanced BC, and prolonged the patients’ PFS and OS [7]. This may be achieved through the so-called “bystander killing” mechanism [47]. Subsequently, the US Food and Drug Administration (FDA) approved T-Dxd as the first targeted therapy for the treatment of patients with unresectable or metastatic HER2-low BC [48]. Since then, the era of binary treatment of HER2 has been broken. DESTINY Break-06 study is another continuous trial for HER2-low metastatic BC designed to evaluate the efficacy and safety of DS-8201 combined chemotherapy in the treatment of HR-positive, HER2-low metastatic BC with failure endocrine therapy. At present, many new ADC drugs (RC48-ADC, ARX788, etc.), BC vaccines (nelipepimut-S, GP2, etc.) and bispecific antibodies (KN026, ZW25, etc.) are being developed, hoping to bring new hope to patients with HER2-low BC [49–52].

This meta-analysis had a few limitations. First, heterogeneity was found in the analysis. However, we used a random effect model to overcome this. Second, the HR value extracted from survival curves may be less reliable than those directly given by authors. Finally, all the articles we included are retrospective studies, which may potentially induce bias in our results.

## Conclusion

In conclusion, our meta-analysis results show that compared with HER2-zero BC, HER2-low BC has a poor response to NACT and a better prognosis in the overall cohort and HR-positive group after NACT. This reminds us that HER-2 low breast cancer has special biological characteristics and requires individualized treatment strategies.

### Electronic supplementary material

Below is the link to the electronic supplementary material.

Supplementary Material 1

Supplementary Material 2

Supplementary Material 3

Supplementary Material 4

## Data Availability

No datasets were generated or analysed during the current study.

## Abbreviations

NACT: Neoadjuvant chemotherapy
BC: Breast cancer
HR-positive: Hormone receptor negative
HR-negative: Hormone receptor positive
HR: Hazard ratio
pCR: Pathological complete response
OS: Overall survival
DFS: Disease-free survival
