# Supplementary material for: Survival outcomes in HER2-low versus HER2-zero breast cancer after neoadjuvant chemotherapy: a meta-analysis
Source: World J Surg Oncol. 2024 Apr 20;22:106. doi: 10.1186/s12957-024-03382-w (PMC11031865; doi:10.1186/s12957-024-03382-w)
Supplement: Supplementary file 2 — Supplementary Material 2 [file 12957_2024_3382_MOESM2_ESM.doc]

| Supplementary Table 1. Detailed search strategy | |
| --- | --- |
| Database | Search strategy |
| Pubmed | (((((breast cancer) OR (breast tumor)) OR (breast neoplasm)) OR (breast carcinoma)) AND ((HER2 low) OR (ERBB2 low)) OR (low HER2)) OR (low ERBB2)))) AND ((neoadjuvant chemotherapy) OR (Neoadjuvant therapy)) |
| Embase | 1.'breast cancer' OR 'breast tumor' OR 'breast neoplasm' OR 'breast carcinoma'  2.'HER2 low' OR 'ERBB2 low' OR 'low HER2' OR 'low ERBB2'  3.'neoadjuvant chemotherapy' OR 'Neoadjuvant therapy'  4. 1 and 2 and 3 |
|  | |
