# Supplementary material for: Survival outcomes in HER2-low versus HER2-zero breast cancer after neoadjuvant chemotherapy: a meta-analysis
Source: World J Surg Oncol. 2024 Apr 20;22:106. doi: 10.1186/s12957-024-03382-w (PMC11031865; doi:10.1186/s12957-024-03382-w)
Supplement: Supplementary file 3 — Supplementary Material 3 [file 12957_2024_3382_MOESM3_ESM.docx]

Supplementary Table 2 Risk of bias in the included cohort studies (by the MINORS quality assessment tool)

|  | **A clearly stated aim** | **Inclusion of consecutive patients** | **Prospective collection of data** | **Endpoints appropriate to the aim of the study** | **Unbiased assessment of the study endpoint** | **Follow-up period appropriate to the aim of the study** | **Loss to follow up less than 5%** | **Prospective calculation of the study size** | **Total quality scores** |  |
| --- | --- | --- | --- | --- | --- | --- | --- | --- | --- | --- |
| Denkert | 2 | 2 | 0 | 2 | 2 | 2 | 2 | 0 | 12 |  |
| Leite | 2 | 2 | 0 | 2 | 2 | 2 | 2 | 0 | 12 |  |
| Domergue | 2 | 2 | 0 | 2 | 2 | 2 | 2 | 0 | 12 |  |
| Alves | 2 | 2 | 0 | 2 | 1 | 1 | 2 | 0 | 10 |  |
| Cosimo | 2 | 2 | 0 | 2 | 2 | 2 | 2 | 0 | 12 |  |
| Shao | 2 | 2 | 0 | 2 | 2 | 2 | 2 | 0 | 12 |  |
| Kang | 2 | 2 | 0 | 2 | 1 | 0 | 2 | 0 | 9 |  |
| Zhou | 2 | 2 | 0 | 2 | 1 | 1 | 2 | 0 | 10 |  |
| Qiao | 2 | 2 | 0 | 2 | 1 | 1 | 2 | 0 | 10 |  |
| Li | 2 | 2 | 0 | 2 | 2 | 2 | 2 | 0 | 12 |  |
| Pöschke | 2 | 2 | 0 | 2 | 2 | 2 | 2 | 0 | 12 |  |
| Li* | 2 | 2 | 0 | 2 | 2 | 2 | 2 | 0 | 12 |  |
| Zhong | 2 | 2 | 0 | 2 | 1 | 0 | 2 | 0 | 9 |  |
| Zhang | 2 | 2 | 0 | 2 | 1 | 1 | 2 | 0 | 10 |  |
