# Supplementary material for: Survival outcomes in HER2-low versus HER2-zero breast cancer after neoadjuvant chemotherapy: a meta-analysis
Source: World J Surg Oncol. 2024 Apr 20;22:106. doi: 10.1186/s12957-024-03382-w (PMC11031865; doi:10.1186/s12957-024-03382-w)
Supplement: Supplementary file 4 — Supplementary Material 4 [file 12957_2024_3382_MOESM4_ESM.docx]

Supplementary Table 3 Results of Begg’s tests for publication bias

| End point(HER2-low VS HER2-zero) | z | *P* |
| --- | --- | --- |
| PCR(All) | 1.85 | 0.064 |
| PCR(HR-positive) | 0.43 | 0.669 |
| PCR(HR-negative) | 0.55 | 0.583 |
| OS(All) | 1.41 | 0.159 |
| OS(HR-positive) | 1.71 | 0.088 |
| OS(HR-negative) | 0.09 | 0.928 |
| DFS(All) | 0.77 | 0.443 |
| DFS(HR-positive) | 0.5 | 0.618 |
| DFS(HR-negative) | 0.49 | 0.644 |
